# Predatory functional responses under increasing temperatures of two life stages of an invasive gecko

**DOI:** 10.1038/s41598-020-67194-0

**Published:** 2020-06-22

**Authors:** Phillip J. Haubrock, Ross N. Cuthbert, Lukáš Veselý, Paride Balzani, Nathan Jay Baker, Jaimie T. A. Dick, Antonín Kouba

**Affiliations:** 1 Senckenberg Research Institute and Natural History Museum Frankfurt, Department of River Ecology and Conservation, Gelnhausen, Germany; 2 University of South Bohemia in České Budějovice, Faculty of Fisheries and Protection of Waters, South Bohemian Research Center of Aquaculture and Biodiversity of Hydrocenoses, Zátiší 728/II, 389 25 Vodňany, Czech Republic; 3 Institute for Global Food Security, School of Biological Sciences, Queen’s University Belfast, 19 Chlorine Gardens, Belfast, BT9 5DL Northern Ireland UK; 4 GEOMAR, Helmholtz-Zentrum für Ozeanforschung Kiel, Düsternbrooker Weg 20, Kiel, Germany; 5 University of Florence, Department of Biology, Via Romana 17, 50121 Florence, Italy

**Keywords:** Ecology, Climate-change ecology

## Abstract

The direct effects of temperature increases and differences among life-history might affect the impacts of native and invasive predators on recipient communities. Comparisons of functional responses can improve our understanding of underlying processes involved in altering species interaction strengths and may predict the effect of species invading new communities. Therefore, we investigated the functional responses of the mourning gecko *Lepidodactylus lugubris* (Duméril & Bibron, 1836) to explore how temperature, body-size and prey density alter gecko predatory impacts in ecosystems. We quantified the functional responses of juvenile and adult geckos in single-predator experiments at 20, 23 and 26 °C. Both displayed saturating Type-II functional responses, but juvenile functional responses and the novel Functional Response Ratio were positively affected by temperature as juvenile attack rates (*a*) increased as a function of increased temperature. Handling times (*h*) tended to shorten at higher temperature for both predator stages. We demonstrate that the effects of temperature on functional responses of geckos differ across ontogeny, perhaps reflecting life-history stages prioritising growth and maturation or body maintenance. This indicates that temperature-dependent gecko predatory impacts will be mediated by population demographics. We advocate further comparisons of functional responses to understand the invasiveness and future predatory impacts of geckos, and other invasive species globally, as temperatures change.

## Introduction

Climate change is pervasive across habitat types and taxonomic groups globally, and effects may interact with other principal drivers, such as habitat loss and invasive species, in altering global biodiversity^1,2^. The interconnection between effects of invasive species and climate change builds upon the preference of numerous species to thrive under warmer temperatures^3^. Further, increasing temperatures may heighten the impact of predatory species on lower trophic levels and, hence, entire ecosystems^4–6^. Therefore, it is crucial to understand how climate change as a driver might affect the behaviour and impact of predatory species, particularly when such species become invasive^7^.

Temperature is considered as one of the most important drivers of interaction strengths and invasive species impacts due to its influence on the feeding, metabolism, and growth of predators and subsequent effects on fitness and behaviour^8,9^. Further, invasive species tend to be more aggressive than their native counterparts^10–13^, consequently leading to higher consumption rates of prey species^14–16^. This linkage between temperature and biological rates is especially true for ectotherms such as amphibians and reptiles. Thus, unsurprisingly, ectothermic invasive species from these groups mostly invade warmer regions in the (sub)-tropics^17^, but with an increase in global temperature, potentially may invade temperate regions as well, particularly given that they are commonly pet traded organisms. Invaded ecosystems are somtimes characterized by a high species diversity, commonly consisting of multiple predatory species that determine the flow of energy within the trophic communities as they share commonly available prey from lower trophic levels^18,19^. Hence, the introduction of an invasive predator could increase top‐down effects via additive feeding combinations with native counterparts, dramatically altering food webs, and thus, potentially increase community suscesptibility towards further invasions, even where an invasive species is competitively disadvantaged^20,21^.

Reptiles are typically predatory^22^, however, the interaction strengths of invasive reptiles towards native prey species remains, to our knowledge, unknown, and thus information on the ecological impacts of these species, especially regarding trophic effects on lower taxa, are urgently required^23^. Moreover, despite the global decline of reptiles^24^, behavioural studies focussing on the feeding ecology of this group across life history stages are generally rare^23,25^, and the potential detrimental effects of invasive reptile introductions on recipient communities have not yet been investigated^26–28^. This may be due to the complexity involved in their laboratory keeping, caring and stocking, species-specific traits and requirements, as well as the protected status of various species^29,30^. However, their unique life-histories and behaviours make them critically important to better understand the mechanisms underlying the invasion processes and the potential impact of invasive reptiles on native biota as climates change.

Lizards of the family Gekkonidae are especially known for their ability for long-distance dispersal due to three primary pathways: (1) hitch-hiking using human mediated means; (2) natural dispersal (e.g. among islands through floating objects), and; (3) through the pet trade^31,32^. One such reptilian species that is infamously known for its adaptable invasive character is the mourning gecko *Lepidodactylus lugubris* (Duméril & Bibron, 1836). In recent decades, it has established self-sustaining populations in suitable habitats of the tropics^32^ with the establishment of various clonal lineages^33^. Its invasion success, despite the rare occurrence of sexual reproduction, is mostly based on its parthenogenetic and thus rapid reproduction rate outgoing from just one individual, with mature females producing a clutch of roughly two eggs every 14–63 days^34,35^. Nevertheless, this species shows a high variability of abiotic tolerances, but detailed information explaining its invasiveness over such broad geographic and climatic scales is unknown^36,37^, especially considering that a substantial variety of lineages thrive in habitats with temperatures below what is considered optimal for foraging^38^. Due to these wide tolerances, *L. lugubris*, as well as other similar species like *Hemidactylus frenatus* Schlegel, 1836, are considered potentially invasive, and their spread and impact may be further exacerbated if current climate predictions are considered^39,40^. In turn, these impacts may be further mediated by demographic characteristics of populations, such as the life-history stages of individual predators.

Quantifications of invasive reptile predatory impacts are lacking or are only anecdotal^23,41^. Functional response studies can be employed to examine the density-dependence of consumer-resource (e.g. predator-prey) interaction strengths. Functional responses quantify resource use as a function of resource density, and functional response types can follow a variety of forms (i.e. Types I, II, III^42,43^). Furthermore, the functional response approach has recently shown great utility in predicting invasive species impacts^44^. For example, Dick *et al*.^45^ illustrated significantly higher impact of the invasive bloody red shrimp *Hemimysis anomala* G. O. Sars, 1907 on prey populations compared to native analogous mysids as revealed by higher functional responses. Similarly, Bollache *et al*.^45^ employed a comparative approach to explore the functional response and hence field impacts of invasive amphipods. More recently, South *et al*.^46^, investigated differences in the functional responses of invasive lionfish *Pterois volitans* (Linnaeus, 1758) under increasing temperatures. With regards to reptiles, Huang *et al*.^47^ investigated the functional response of the female Mongolia racerunner *Eremias argus* Peters, 1936 on the Asian grasshopper species *Oedaleus asiaticus* Bey-Bienko, 1941, in the context of biocontrol. However, functional response studies are needed for a variety of native and invasive reptiles, specifically geckos, to help explain and predict their impacts^47,48^. Indeed, information on underlying processes and biological responses to climate change are lacking for the vast majority of reptilian species^49,50^. Thus, the usage of *L. lugubris* in functional response experiments can permit the analysis of the thermal plasticity of this species and associated behavioural responses, considering anticipated future climate conditions, and therefore any temperature-dependent predatory impact. Moreover, such an approach may reveal the impacts similar species could have on prey populations, and could test if responses are contingent on population demographics^40^.

Thus, we hypothesized that interaction strengths of reptiles are responsive to temperature, increasing their impact globally under warming scenarios^40^. More specifically, we predicted that functional responses of the invasive gecko *L. lugubris* increase with temperature due to their ecotothermic nature^38,51^. Furthermore, we ask whether feeding and predation impacts differ across predator life stages, which could be another key success factor for invasive reptiles apart from reproductive strategies and high abiotic tolerances^52^. We hypothesise that both juvenile and aduilt geckos will respond similarly to increasing temperatures with respect to their prey attack rates (*a*) and handling times (*h*) and the novel FRR metric (Functional Response Rato, *a/h*; see Methods). To test our hypothesis, we investigated the joint effects of temperature and life stage (juveniles and adults) on the functional responses of this widely distributed invasive gecko.

## Results

**Figure 1:**
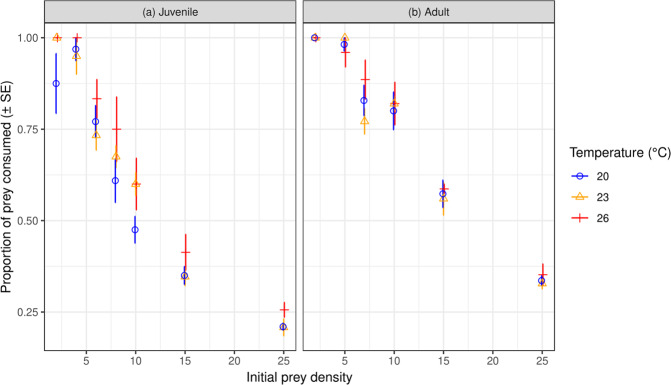
Proportion of prey consumed by both life stages of *Lepidodactylus lugubris* across temperatures and initial prey densities.

For juvenile *L. lugubris*, proportional prey consumption was significantly affected by temperature (χ^2^ = 6.16, df = 2, *p* < 0.05) (Fig. 1a), with consumption significantly greater at the 26 °C compared to 20 °C treatments (*p* < 0.05); other pairwise temperature comparisons were non-significant (*p* > 0.05). Proportional consumption also related significantly negatively with increasing prey density (χ^2^ = 283.68, df = 1, *p* < 0.001), and there was no significant interaction term (χ^2^ = 0.48, df = 2, *p* > 0.05). Contrastingly, for adults, proportional prey consumption was not significantly affected by temperature (χ^2^ = 0.56, df = 2, *p* > 0.05) (Fig. 1b), and again decreased significantly with increasing prey density (χ^2^ = 315.85, df = 1, *p* < 0.001) and there was no significant interaction term (χ^2^ = 0.03, df = 2, *p* = 0.99).

**Figure 2:**
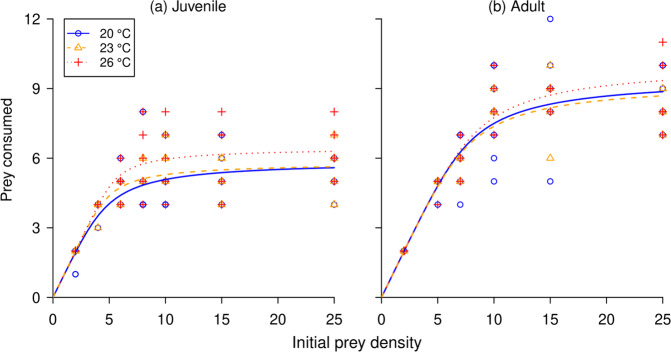
Functional responses of juvenile and adult *Lepidodactylus lugubris* across three temperature treatments. Points are raw underlying data.

Significantly negative linear coefficients indicated that both juvenile and adult *L. lugubris* life stages exhibited Type II functional responses across all temperatures (Table 1; Fig. 2). For juveniles, attack rates strongly trended to increase with temperature, whilst attack rates of adults were very consistent across temperatures (Table 2). However, there were no significant pairwise comparisons among temperatures within each life stage (Table 3). For both juveniles and adults, handling times generally shortened at the highest temperature compared to the lowest temperature, in turn driving higher maximum feeding rates (Table 2); however, again, there were no significant pairwise differences in handling times *h* within life stages (Table 3).

**Table 1:** Linear coefficients resulting from logistic regression considering proportional prey consumption as a function of prey density aross all *Lepidodactylus lugubris* life stage and temperature treatment groups.

Life-stage	Temperature (°C)	Linear coefficient	*p*-value
Juvenile	20	–0.13	<0.001
Juvenile	23	–0.14	<0.001
Juvenile	26	–0.14	<0.001
Adult	20	–0.14	<0.001
Adult	23	–0.14	<0.001
Adult	26	–0.15	<0.001

**Table 2:** Attack rate, handling time and maximum feeding rate estimates resulting from Rogers’ random predator equation, alongside functional response ratios, across all *Lepidodactylus lugubris* life stage and temperature treatments.

Life-stage	Temperature (°C)	Attack rate (*a*), *p*-value	Handling time (*h*), *p*-value	Maximum feeding rate (1/*h*)	Functional response ratio (*a*/*h*)
Juvenile	20	5.27, <0.001	0.17, <0.001	5.87	30.94
Juvenile	23	8.52, <0.05	0.17, <0.001	5.80	49.45
Juvenile	26	11.67, >0.05	0.16, <0.001	6.45	75.29
Adult	20	6.58, <0.001	0.11, <0.001	9.50	62.54
Adult	23	6.43, <0.01	0.11, <0.001	9.31	59.83
Adult	26	6.47, <0.001	0.10, <0.001	10.07	65.15

**Table 3:** Pairwise comparison coefficients resulting from the difference method between temperatures within each *Lepidodactylus lugubris* life stage. Significance is tested against Bonferroni-corrected α to account for multiplicity within each functional response parameter (attack rate, *a*; handling time, *h*).

Life-stage	Temperature comparison (°C)	Parameter	*z*-value	*p*-value
Juvenile	20–23	*a*	0.79	>0.05
Juvenile	20–26	*a*	0.94	>0.05
Juvenile	23–26	*a*	0.41	>0.05
Juvenile	20–23	*h*	0.09	>0.05
Juvenile	20–26	*h*	0.77	>0.05
Juvenile	23–26	*h*	0.76	>0.05
Adult	20–23	*a*	0.06	>0.05
Adult	20–26	*a*	0.05	>0.05
Adult	23–26	*a*	0.02	>0.05
Adult	20–23	*h*	0.16	>0.05
Adult	20–26	*h*	0.48	>0.05
Adult	23–26	*h*	0.54	>0.05

**Figure 3:**
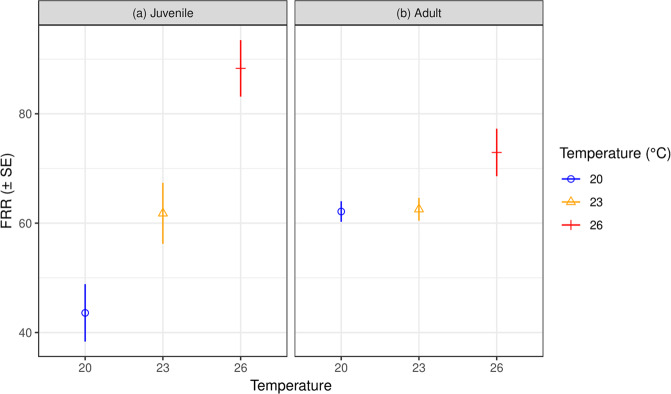
Functional response ratios (FRR; *a*/*h*) of both life stages of *Lepidodactylus lugubris* across temperatures, resulting from bootstrapped functional response parameters (*n* = 30 per experimental group).

However, Functional Response Ratios [FRRs^53^], which capture both the generally increasing attack rates and decreasing handling times above, clearly increased with increasing temperature for juveniles, but not for adults (Table 2; Figs. 2, 3). FRRs were therefore significantly affected by warming in juveniles (χ^2^ = 41.79, df = 2, *p* < 0.001), however, not in adults (χ^2^ = 3.62, df = 2, *p* = 0.16) (Fig. 3). At the juvenile stage, significantly greater FRRs ensued following each incremental temperature increase (all *p* < 0.01).

## Discussion

Understanding the implications of climate change for biotic interaction strengths is critical for predictions of community dynamics under global environmental change scenarios. Warming may exacerbate the ecological impacts of invasive species in driving biodiversity loss; however, these effects may, in turn, be dependent on the demographic characteristics of predator populations. The present study demonstrates temperature-dependences of the functional response of an understudied invasive reptile, which previously lacked quantifications of interaction strengths. However, temperature effects were also contingent on predator life stages, with juvenile predatory performance significantly heightened by warming, whilst adult performance was consistent. Functional response studies on single species have been found to be useful predictive measures of their in-field ecological impacts, and particularly in respect to the measurement of relevant context-dependencies^44^. Therefore, although focused on a model predator-prey system, our findings are of potential broad relevance to both climatic and ontogenic effects on invasive species impacts, particularly given that predicting the effects of climate change on species interaction strengths with certainty is highly challenging.

Changes in temperature due to, for example, climate change can have various effects on predator behaviour and demographics. In endothermic species generally, temperature has been shown to directly affect functional response parameters, i.e. attack rate and handling time, making them temperature-dependent^54^. However, reptiles, and especially geckos, are a relatively neglected group in terms of temperature-dependent assessments of impact, particularly in an invasions context, despite the recently increased focus on temperature effects on the physiological activity of this group^55^. Although a universal temperature dependence has been postulated for all ectothermic animals^8^, this is seemingly not the case in regard to the attack rate and handling time in ectothermic reptiles^41^. Similarly, our results indicate that functional responses of geckos are only significantly affected by temperature in some life stages. Attack rates and handling times were not significantly different when considered singularly for either life stage. However, when the two parameters were amalgamated in the novel FRR (i.e. *a/h*), which considers the joint effects of the attack rate and handling time parameters^53^, we found clear and significant increases in species interation strength for juveniles but not adults over temperature increases. Accordingly, ontogenic stage is clearly an important factor determining ecological impacts of invasive species: while both life stages expressed the same functional response type (II), juveniles expressed a significantly larger FRR, i.e. direct feeding impact, under higher temperatures, whilst adults were not significantly affected yet relatively high across temperatures. The use of the novel FRR metric can thus strengthen comparative assessments of the influence of temperature and other context-dependencies on ecological impacts.

The impacts of invasive reptiles on native species under shifting environmental contexts have not yet been investigated. Accordingly, so far, the direct effects of increasing temperatures on species are mostly unknown^23,24^ and comparative functional response studies for reptiles, native or invasive, are scarce^47,48^. Consequently, functional response data for reptiles are not available and, thus, impact predictions cannot be applied^56^. Therefore, functional response experiments with reptililes, especially gekkonid species, have to be performed to address knowledge gaps in biological invasions and trophic interactions^44,57^. In this regard, especially the Type II functional response identified here, brings further implications, as high predation rates at low prey densities may drive prey to extirpation owing to a lack of prey refuge. Prey-specific reponses to changing climate are also of importance, as if a given prey responds to warming by increasing its abundance, higher predatory impacts by invasive species may be remediated^58^. As such, this requires further system-specific investigation to decipher holistic responses of predator-prey participants to environmental changes. Understanding and considering such context-dependencies associated with environmental change and in regard to increasing global temperatures is crucial for the prediction of impact. This can be especially accomplished using empirical experiments that consider differences in temperature, in turn potentially explaining and quantifying impacts of invasive predators in novel ranges^45,56^. The findings of this study therefore support the documented feeding impacts of *L. lugubris* in tropical and subtropical regions, as well as other regions where the species is yet to invade.

Nowak^41^ showed that ectothermic vipers expressed lower functional responses due to longer prey handling times, higher efficiency in food conversion, as well as a reduced ability to respond to short-term changes in prey abundances compared to endothermic species. In the present study, we identified a general trend in attack rates as well as handling times among temperature for juvenile *L. lugubris*. Hence, the findings of Nowak^41^, i.e. the lower temperature-dependence for attack rate and handling time in ectothermic reptiles than in endothermic animals, might be representative for adult *L. lugubris* used in this study and potentially reptiles in general, explaining the observed differences in FRRs among life stages. As a result, it can be assumed that these differences originate from physiological processes, i.e. the variable food conversions, and thus food consumption needed by juveniles, as energy is invested in growth rather than reproduction^23,25,41^. Considering the rapid growth and maturation in juvenile *L. lugubris*^35,38^, it is possible that increased food consumption is needed at higher temperatures, while adults only need to maintain body weight and reproductive status^35^.

Moreover, juveniles and adults show slight phenotypic differences in their colouration patterns, but also vary in their activity time (i.e. adults are considered to be mostly nocturnal, while juveniles do not exhibit clear patterns). However, despite this species being considered a nocturnally active insectivore^59^, adults have been shown to be very adaptive to the environments they inhabit^60,61^. More specifically, *L. lugubris* shows the potential to adapt to anthropogenic activity and stressors (e.g. artificial light sources^37^) and adjust its feeding activity and activity patterns by predating close to these light sources^32,62,63^. The difference in activity time^33,59^, considering the similar feeding response behaviour, could be a potential intra-species avoidance mechanism, supporting thepotential of this species to establish and become invasive. Indeed, as the current distribution of this species substantially derives from the pet trade, it can be assumed that these organisms are accustomed to anthropogenic disturbances.

Furthermore, the invasive capacity of *L. lugubris* is reinforced by female reproductive output, with a clutch laid every 14–63 days^51^, and with an even more accelerated reproduction rate when population density is low^51,61,64^, compounded by the rapid growth rate in hatchlings (size of hatchlings: snout vent length SVL: 15–22 mm, TL: 32–44 mm^65^). As functional responses only asses the *per capita* effects of consumers, further effects of feeding rates on e.g. abundance due to increased fecundity are important^7,56^. In essence, species with high functional responses could have low abundances, and thus could have small population-level impact on prey, whilst species with low functional responses, but relatively high abundances, could have higher impact. Such combination of functional responses and abundances has been shown to correlate tightly with known invader ecological impacts^7^. In addition, understandings of temperature effects on other beaviours of geckos, as well as resource availability and composition, are inherently important. Effects of temperature extremes on foraging rates of this species also require consideration to better understand the implications of ongoing climate change^66^.

As a nocturnal species, *L. lugubris* can, despite its laboratory thermal preference of 29.2 °C which reflects a commonly 3–4 °C colder normal field temperature^38^, actively forage and sprint at temperatures that are considered as below optimal. Moreover, the temperature range employed in this study may be better representative of non-native regions, indicating that the species can have high predatory impacts in suboptimal conditions. Accordingly, our results may be viewed as conservative in this regard. Therefore, and in respect to global warming, *L. lugubris* might become more dominant over its current competitors as Bolger & Case^37^ were able to show significant variability in thermal biology of different *L. lugubris* clone lineages that evolved within considerably short periods. While we used specimens identified as clone lineage A, the most common clone lineage in the pet trade, it can be assumed that other lineages might show differences in functional responses and climatic variation due to inter-clonal differences in spatial and temporal distribution^37^, and potential differences in SVL^60^. Further studies on other clonal lineages as well as a wider temperature gradient are therefore needed to fully understand the differing functional response behaviour of juvenile and adult *L. lugubris*, both inside and outside of thethermal optimum of this species. Moreover, these clone lineages exert differences in “boldness”^63^ and thus willingness to risk hunting in open space, and thus its close association with humans might give this species an advantage in the future. In addition, as our study used animals traded in captivity, examination of wild populations in other geographic regions (invasive and native) would provide further information on interaction strengths of wild individuals^67^.

Considering its vast distribution and presence in the pet trade, *L. lugubris* is prone to occur in the wild outside of its natural range^33,63^. However, while it seems to suffer from competition with other gecko species^68^, its ongoing spread and capacity to become invasive seem mostly limited by climatic conditions^37^. Nevertheless, considering its ability to reproduce asexually, exert higher functional responses at higher temperatures and spread through human pathways, it could become a potential future invader in current climatically unfavourable regions^69^. Moreover, the occurrence of milder winters may facilitate its establishment even in greater latitudes, into temperate zones.

In conclusion, we show that the temperature-dependence of the functional response in the mourning gecko *L. lugubris* is, in turn, dependent on life stage, with juveniles but not adults showing increased functional responses as temperatures increase. The novel Functional Response Ratio, FRR, by combining attack rates and handling times, clearly helps resolve such effects. Further, the success and impacts of this species might vary with its population demographics, and combining *per capita* effects with its abundance, and other life history traits, will enable future predictions of invasiveness under climate change.

## Methods

Experiments were conducted using the mourning gecko *Lepidodactyus lugubris* (Gekkonidae), at juvenile and adult life stages, preying on the adult bean weevil *Acanthoscelides obtectus* (Say, 1831) under three temperature regimes (20, 23 and 26 °C; see below). *Lepidodactylus lugubris* is a parthenogenetic gecko native to the Arno Atoll, Marshall Islands^70^, and insects likely comprise an important dietary component^71^. Currently, geckos are distributed in almost all tropical and subtropical regions around the world^72^. Individuals can reach a total length of 8–10 cm, but tend to remain around 7–8 cm in captivity or sub-optimal conditions^35,73^. The clonal identity of experimental specimens was determined according to Ineich & Ota^60^ and Griffing *et al*.^33^. Only adult individuals (and their respective offspring) identified as clone lineage A were chosen as it is the most common lineage found in laboratories and within the pet trade. The prey, *A. obtectus* is a weevil species native to North America but now ubiquitously distributed due to global trade. As such, we believe it to be a representative prey for this study, particularly considering the observed diet of *L. lugubris* that consists of ground dwelling amphipods and insects^71^. Specimens reach an average size of 3–4 mm and this species was also chosen as potential prey due to its similarity in size to various native coleopteran species (size, colour, movement speed^74,75^), and it is commonly used to feed captive geckos as well as relatively easy to care for and rapidly cultivate^76^.

Individuals of *L. lugubris* were obtained from private keepers and housed individually in cylindrical enclosures (diameter 30 cm; height 15 cm; ~10.6 L). These enclosures were also used as experimental arenas to minimize the stress associated with relocating individuals into novel experimental enclosures. As juveniles and adults were thus not in enclosures scaled to account for their differing body sizes, we compare feeding results only within each life stage and do not compare statistically between life stages (see below). Specimens of *A. obtectus* were bought online from the pet trade, as they are a common food item used by gecko keepers, and cultured following Leroi^76^. We ensured that prey specimens were at least 3 mm large. Juvenile geckos had a total length of 40 ± 4 mm (Snout-Vent-Length SVL: 23 ± 3 mm) and adults 67 ± 5 mm (SVL: 51 ± 4 mm). Although we used organisms that were not sourced from the wild, the pet trade is a key potential pathway through which this and other invasive reptiles could be introduced. Therefore, our use of captive organisms is empirically relevant, as organisms from captivity could potentilly establish viable populations in the wild following release. Adult maturity was assumed when individuals were approximately 41 mm SVL, following Messenger^77^ and Limpus *et al*.^78^. Before the experiments, predators and prey were acclimated and maintained at room temperature (20 °C) for 2 weeks to ensure that predators were not gravid or shedding. For trials at increased room temperatures (23 and 26 °C), the same acclimatization period of 2 weeks was used, with an increase of 1 °C or 2 °C, respectively, every 2 days to reach 23 °C or 26 °C, followed by a final period of 8 days at the nominal final temperature. These ambient temperatures were chosen to reflect different conditions, i.e. the temperature gradient at which this species occurs and actively predates under consideration of possible variation between day and night time^35,72^. Moreover, according to the “BNA Bundesverband für fachgerechten Natur- und Artenschutz e.V.” derived from Directive 2010/63/EU of the European Parliament and of the Council (article 1) on the protection of animals used for scientific purposes as well as § 1 of the “Tierschutzgesetz”, artificial stress through e.g. constantly high temperature during experimentation should be avoided. Prior to the experiments, all predator individuals were fed in excess with adult *Drosophila melanogaster* Meigen, 1830. Before each experimental trial, *L. lugubris* specimens were randomly selected and starved for 24 hours to standardise hunger levels^79^. All experiments were conducted under 12 h light: 12 h dark cycle.

Functional responses for juvenile and adult *L. lugubris* were quantified at three temperatures (20, 23 and 26 °C) for a time period of 24 h. We examined functional responses phenomenologically, that is, employed a comparative and factorial experimental design to compare feeding rates across standardised experimental conditions, without mechanistically validating feeding parameters^44^. Pilot studies were used to indicate experimental prey densities that: (1) resolve the shape of the functional response curve at low prey densities, and; (2) result in an functional response curve asymptote at high prey densities. Hence, juveniles were given seven prey densities (2, 4, 6, 8, 10, 15, 25 individuals enclosure^–1^) and adults 6 prey densities (2, 5, 7, 10, 15, 25 individuals enclosure^–1^). Functional response studies require a range of prey densities to be provided, which capture a range of potential empirical prey scenarios that a consumer could encounter. In turn, parameterisation allows for examination of both low- and high-prey density effects by consumers, with implications for prey population stability. Prey were added into the respective predator enclosures at the beginning of the light cycle. At 20 °C, eight replicates were conducted for juvenile *L. lugubris* and 11 replicates for adults, for each prey density. At 23 and 26 °C, five replicates were conducted for both life stages at the respective prey density. Nevertheless, each individual of *L. lugubris* was only used in one experimental trial, (i.e. one individual was used for all respective prey densities in the specific trial). In total, we thus used 21 adults and 18 juveniles. Between each change in prey density, specimens were left in solitude for 48 hours and not fed for the 24 hours prior to the change in feeding density. The natural background mortality for *A. obtectus* at each density and every temperature was investigated without the presence of a predator in controls (n = 10). As there was absolute survival in these controls (i.e. treatments without predators), data did not need corrected for background prey mortality. Juvenile and adult gecko stages were tested separately due to logistical reasons, and in the same sized experimental arenas to avoid stress of moving geckos to novel arenas for experimentation. Given that cage size was not scaled to predator size, and subtly different prey densities were used for each predator type, the predator life stages were not directly comparable statistically.

Consequently, separate binomial generalised linear models with logit links were used to examine the influence of temperature and prey density on proportional prey consumption for each predator life stage. There was no evidence for residual overdispersion^80^. The initial models included both single and interacting terms, with non-significant terms removed stepwise to obtain the minimum adequate model in each case. Accordingly, the final model included only terms with significant *p*-values. Final model selection was further confirmed by derivations of Akaike’s information criterion, adjusted for small sample sizes (lower values indicate a better fit). Analyses of deviance were used to infer main effect significance levels, with Tukey-style *post*-*hoc* tests performed for pairwise comparisons where necessary.

For each temperature and predator life stage treatment, logistic regression was performed to examine the relationship between the initial prey density and the proportion of prey consumed, and thus identify the shape of the functional response curve. A Type II functional response is inferred in the presence of a significantly negative linear coefficient, where the proportion of consumed prey declines monotonically (i.e. a unidirectional decrease) with the initial density of prey. Conversely, a significantly positive linear coefficient and significantly negative quadratic coefficient indicates a Type III functional response^81^. We applied the Type II Rogers’ random predator equation^82^ to account for prey depletion during the experiments:

1 $${N}_{e}={N}_{0}(1-\exp (a({N}_{e}h-T)$$

where *N*_e_ is the number of prey eaten, *N*_0_ the initial prey density, *a* the predator attack rate (classically interpreted as the search efficiency), *h* the predator handling time (defined as the time spent pursuing, subduing, and consuming each prey item plus the time spent preparing to search for the next prey item), and *T* the duration of the experiment. To fit Rogers’ model to the experimental data, Lambert’s *W* function was implemented in the “*emdbook*” R package^83–85^. Following^81^, we employed the difference (delta) method to compare functional response attack rate and handling time parameter estimates across temperatures, separately for each life stage. We used a Bonferroni correction of α to account for multiple pairwise testing among the temperature treatments (i.e. α = 0.017). To amalgamate and further compare the functional response parameters *a* and *h* among temperatures and life stages, the functional response ratio (FRR^53^) was estimated using the attack rate *a* divided by the handling time *h*, based on 30 non-parametric bootstraps of each parameter within all treatments. The FRR has advantages due to combining both *a* and *h*, as high values for *a* and low values for *h* would result in high predatory impacts. One-way Kruskal-Wallis rank sum tests were used to test whether FRRs differed across temperatures within each ontogenic stage. Dunn tests were used *post*-*hoc* with Bonferroni corrections to account for comparison multiplicity.

All specimens were obtained from the private collection of one of the authors and no individual was sacrificed. Special permissions were not required for experimentation based on evaluation by the Department V 54 - Veterinary and Consumer Protection in the Regional Council of Darmstadt. Animal housing conditions complied with the guidelines of the “BNA Bundesverband für fachgerechten Natur- und Artenschutz e.V.” derived from Directive 2010/63/EU of the European Parliament and of the Council (article 1) on the protection of animals used for scientific purposes. Experimental protocols were not invasive and therefore complied with § 1 of the “Tierschutzgesetz”.
